# Correlation of central corneal thickness and optic nerve head topography in patients with primary open-angle glaucoma

**DOI:** 10.4103/0974-620X.64231

**Published:** 2010

**Authors:** Tharwat H. Mokbel, Asaad A. Ghanem

**Affiliations:** Department of ophthalmology, Mansoura University Ophthalmic Center, Mansoura, Egypt

**Keywords:** Central corneal thickness, optic nerve head, primary open angle glaucoma, visual field

## Abstract

**Purpose:**

To evaluate whether changes in optic nerve head topography and visual field in patients with primary open-angle (POAG) are related to central corneal thickness (CCT).

**Materials and Methods:**

Eighty eyes of 50 patients with POAG underwent ophthalmic examination; optic nerve head imaging with the Heidelberg Retina Tomography II (HRT II), ultrasound corneal pachymetry, and visual field evaluation with the Humphrey visual field analyser (program 24–2). Correlation between CCT, age, gender, family history of glaucoma, visual acuity, intraocular pressure (IOP), optic disc surface area, vertical and horizontal cup: disc ratios, neuroretinal rim area, mean deviation of visual field, and number of glaucoma medications was analyzed. Patients were divided into a thin CCT group <540 μm or a thick CCT group >540 μm. Pearson correlation was used for correlation coefficient and a *P* value of <0.05 was considered statistically significant.

**Results:**

Thin CCT was significantly correlated with vertical and horizontal cup: disc ratios, neuroretinal rim area loss, and smaller optic disc surface area (r=0.043, r=0.021, r=0.036, and 0.031 respectively). Thin CCT was also significantly associated with worsened mean deviation of visual field, and increased number of glaucoma medications (r=0.065 and r=0.423). Patients with positive family history correlated with with greater vertical cup: disc ratio, and more glaucoma medications but this was not statistically significant.

**Conclusions:**

In patients with POAG those with thinner CCT are likely to develop greater glaucomatous optic nerve and visual field damages than those with a thicker CCT.

## Introduction

Central corneal thickness has recently been shown to be an important risk factor for the development and severity of glaucoma.[1] Nowdays, the management strategy is directly affected, by CCT measurement in 15% of the glaucoma patients.[2] Corneal thickness decreases through infancy and reaches adult thickness between the ages of 2–4 years.[3]

It is unclear whether risk attributed to CCT is only the result of inaccuracies in measurement of IOP or whether there are additional related factors, such as properties of the posterior sclera and lamina cribrosa, which may significantly influence the development and progression of glaucoma.

Several reports have focused on the concern that thinner than- average corneas may underestimate the true IOP whereas thickener than average corneas may overestimate the true IOP. This effect has been found to be in the order of 1 mmHg correction for every 25 μm deviation from a CCT of 550 μm.[4]

Central corneal thickness has recently been recognized as a significant risk factor for progression of ocular hypertension to primary open-angle glaucoma in the Ocular Hypertension Treatment Study.[5] This study was the first to prospectively demonstrate that a thinner CCT predicts the development of POAG. They found that a decrease in CCT of 40μm added a 70% increase in risk.

In OAG and OHT, a thin cornea is more strongly associated with disease severity than IOP.[1] However, CCT has a significant effect on IOP measured by applanation tonometry. Some cannulation studies have shown tonometric estimates to be lower than actual IOP in eyes with thinner corneas and vice versa, indicating that CCT can have significant influence in the risk assessment for open-angle glaucoma based on applanation tnometry measurement.[6]

In most patients with glaucoma, the central lamina cribrosa is covered by little or no neural or glial tissue. Thus, topographic images of optic nerve head and peripapillary retina obtained by scanning laser ophthalmoscope in POAG, provide us with high sensitivity and specificity quantitative association.[7]

Visual field testing is an essential tool for diagnosis and follow up of open-angle glaucoma. The Humphrey Visual Field Analyzer provides several global indices: mean deviation (MD) and corrected pattern standard deviation (CPSD) which are calculated by the StatPac program and do not depend on the any observer input. MD index estimates the uniform part of the visual field deviation and is influenced by a generalized depression (e.g cataract). CPSD index estimates the non-uniform part of the visual field deviation; it is affected by localized defects. Also, CPSD represents pattern standard deviation index (PSD) adjusted for short-term fluctuation (SF) index.[8]

In the current study, we examined the relationship between central corneal thickness and incident changes in the optic nerve head and visual field in POAG patients.

## Materials and Methods

### Study design

This was a cross-sectional study. After explaining the details of the study, we obtained written informed consent from all patients before enrollment. The study was approved by Mansoura university hospital trust ethics committee and was carried out in accordance with the Declaration of Helsinki (1989) of the world medical association.

### Patients

Fifty patients (27 males and 23 females) with medically controlled primary open-angle glaucoma which attended the outpatient clinic of Mansoura Ophthalmic Center were included in this study. Patients with POAG had gonioscopically open angle and fulfilled at least two of the following criteria:characteristic glaucomatous visual field defects, glaucomatous optic neuropathy, and /or IOP > 21 mmHg. The IOP measurement was > 21mmHg on at least three occasions.

### The selected patients were classified into:[9]

Group I: included 42 eyes (25 patients) with a thick CCT (≥ 540 μm);group II: included 38 eyes (25 patients) with a thin CCT (< 540 μm).

Each patient underwent a comprehensive ophthalmologic examination, including review of medical history, best-corrected visual acuity, slit-lamp biomicroscopy, IOP measurement using Goldmann applanation tonometry, gonioscopy, automated perimetry using Humphrey 24–2 visual field analyzer, CCT measurement using ultrasonic pachymetry, and optic nerve head topography using Heidelberg Retina Tomograph (HRT II) (HRT II; Heidelberg Instruments, Heidelberg, Germany).

### Inclusion criteria

Inclusion criteria included age ≥ 40 years;best- corrected visual acuity not worse than logMAR 0.4, spherical refractive errors ≤ ±5.00 and cylinder ≤ ±3.00 diopters, reliable Humphrey visual fields, and good image quality with HRT.

### Exclusion criteria

Exclusion criteria were: concomitant corneal or retinal diseases, systemic diseases known to affect the visual field (e.g demyelinating disease and pituitary lesions), contact lens wear (should not have been worn for at least 3 week before examination), history of intraocular surgery, secondary causes of glaucoma (including pseudoexfoliation, pigment dispersion glaucoma, iridocyclitis, and trauma), and retinal laser procedures (including panretinal photocoagulation).

### Central corneal thickness evaluation

Central corneal thickness (CCT) was measured with ultrasonic pachymetry (Sonoscan, model 4000 AP). The pachymetry measurement recorded for each eye separately was the mean of three measurements.

### Optic nerve head topography evaluation

Optic nerve head topography was imaged with confocal scanning laser ophthalmoscope (Heidelberg Retina Tomograph II)

The topography image was computed from the three dimensional image, then the contour line was drawn at the inner edge of the scleral (Elschnig’s) ring. The reference plane was automatically determined parallel to the peripapillary retinal surface and located 50 μm under the retinal surface at the contour line and on the papillomacular bundle (350° to 356°). Patients whose HRT II images had a standard deviation ≤ 50 (sufficient quality) were included for final analysis.

The optic nerve head parameters measured by HRT II and used in our study were optic disc area, cup area, rim area, vertical cup: disc ratio, and horizontal cup: disc ratio.

### Visual field evaluation

Global indices (MD, PSD) of the visual field were measured by the Humphrey field analyser (Humphrey-Zeiss, Dublin, CA, USA) using the full threshold 24–2 program. Reliable Humphrey automated perimetry was defined by having fewer than two of the following characteristics: fixation losses greater than 20%, false positive responses greater than 33%,or false negative responses greater than 33%. Glaucomatous visual field loss was defined as two consecutive reliable abnormal visual fields with a pattern standard deviation outside 95% normal limit, or a glaucoma hemifield test outside 99% normal limit, or three or more adjacent points with *P* <5% on the pattern deviation probability plot of which one must have *P* <1%.[10]

### Statistical analysis

This was done using SPSS program (standard version 10, 1999). Values were recorded as mean±SD. Statistical significance between groups was determined using unpaired student-t-test for comparing means of quantitative data. Pearson correlation (r) was used for correlation coefficient. A *P* value of <0.05 was considered to be statistically significant.

## Results

The study included 80 eyes of 50 patients with medically controlled POAG (27 males and 23 females). The range of age was 42-68 years. Patients were divided to thick group or to thin group based on their median central corneal thickness (CCT).

Table 1, shows the demographic characteristics of the included POAG patients. There were no significant differences in age, gender, positive family history, diabetes mellitus, systemic hypertension, migraine, and vascular disorders between the two groups (*P*=0.65, *P*= 0.56, *P*=0.21, *P*=0.34, *P*= 0.45, *P*= 0.52 and *P*= 0.37, respectively, unpaired t test)

**Table 1:** Demographic characteristics of POAG patients

	*Thick CCT group (n=25)*	*Thin CCT group (n=25)*
Eyes (n)	42	38
Right / Left eyes (n)	22 / 20	20 / 18
Age (yrs)	62.2 ±15.6	63.2 ±12.5
Gender (M:F)	14:11	13:12
Positive family history	8 (32)	6 (24)
Diabetes mellitus	5 (20)	6 (24)
Systemic hypertension	9 (36)	7 (28)
Migraine	4 (16)	5 (20)
Vascular disorders	3 (12)	2 (8)

CCT = Central corneal thickness, N= number, Yrs= years, M:F= Male: Female, Figures in parentheses are in percentage

The ophthalmic characteristics of POAG patients are shown in Table 2. Only CCT differed statistically between both groups (*P* <0.05).

**Table 2:** Ophthalmic characteristics of POAG patients

	*Thick CCT group (n=25)*	*Thin CCT group (n=25)*	P* value
Spherical equivalent (D)	0.7±3.5	0.62±2.1	0.85
Visual acuity (LogMar unit)	0.35±0.15	0.27±0.12	0.24
Intraocular pressure (mmHg)	26.6±4.5	27.5±6.5	0.68
Central corneal thickness (μm)	576±36.1	518±34.2	< 0.05
Optic disc area (mm^2^)	2.13±0.31	1.95±0.29	0.25
Cup area (mm^2^)	0.23±0.15	0.48±0.12	0.15
Rim area (mm^2^)	1.27±0.51	1.15±0.28	0.58
Vertical cup: disc ratio	0.64±0.21	0.67±0.31	0.48
Horizontal cup: disc ratio	0.61±0.24	0.63±0.27	0.57
Mean deviation (dB)	−6.78±1.62	−6.87±1.45	0.37
Pattern standard deviation (dB)	4.87±1.28	5.65±0.39	0.61
Number of medications	1.2±1.5	1.8±1.3	0.63

CCT = Central corneal thickness; HRT= Heidelberg retina tomograph; dB= decibels; D= diopters; mmHg=millimeters of mercury; LogMar= Logarithm of the minimum angle of resolution

* Results of the unpaired t test

Table 3 shows the correlation coefficients regarding relationship to CCT. Regarding optic nerve head parameters by HRT, there was statistically significant correlation between thin CCT and optic disc area (r = - 0.251, *P* = 0.031), neuroretinal rim area (r = 0.036, *P* = 0.016), vertical cup: disc ratio (r = 0.043, *P* = 0.014), and horizontal cup: disc ratio (r = 0.031, *P* = 0.021). Regarding the visual field there was only a statistically significant correlation between thin CCT and mean deviation visual field (r =- 0.065, *P* = 0.003). In addition, there was significant correlation between thin CCT and number of glaucoma medications (r = 0.423, *P* = 0.021).

**Table 3:** Correlation coefficients regarding relationship to thin central corneal thickness

	*Thin CCT group*
Optic disc area (mm^2^)	−0.251 (<0.05)
Cup area (mm^2^)	−0.215 (>0.05)
Rim area (mm^2^)	0.036 (<0.05)
Vertical cup: disc ratio	0.043 (<0.05)
Horizontal cup: disc ratio	0.031 (<0.05)
Mean deviation (dB)	−0.065 (<0.05)
Pattern standard deviation (dB)	0.365 (>0.05)
Number of medications	0.423 (<0.05)

dB= decibels, mm=millimeter, CCT = Central corneal thickness

Table 4 shows the correlation coefficient between visual field mean deviation and topographic optic nerve head parameters by HRT. There was statistically significant correlation between mean deviation and optic disc area (r = - 0.425, *P* = 0.015), neuroretinal rim area (r = 0.528, *P* = 0.031). There was no statistically correlation between mean deviation and cup area (r=- 0.217, *P*= 0.165), vertical cup: disc ratio (r=0.148, *P*=0.112), horizontal cup: disc ratio (r = 0.136, *P* =0.135).

**Figure 1:**
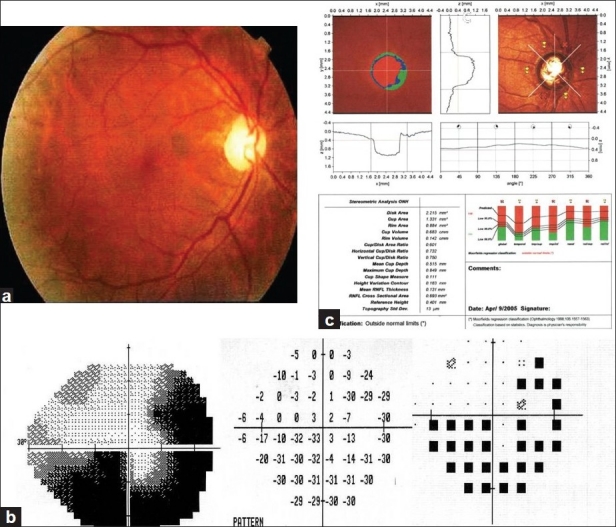
Female 55 years with primary open angle glaucoma showing (a) right optic disc photography with cup:disc ratio=0.7, (b) inferior altitudinal visual field defect with superior temporal arcuate schotoma, (c) HRT outside normal limits

Figure 1 displays a case with POAG showing fundus photography, visual field defect, and optic nerve head topography.

**Table 4:** Correlation coefficients between visual field mean deviation and topographic optic nerve head parameters by HRT

*Parameters*	*r*	*P*
MD and disc area	−0.425	<0.05*
MD and rim area	0.528	<0.05*
MD and cup area	−0.217	0.165
MD and vertical cup disc ratio	0.148	0.112
MD and horizontal cup disc ratio	0.136	0.135

HRT= Heidelberg retina tomograph; MD= Mean deviation

* Pearson correlation coefficient

## Discussion

Glaucoma is a progressive optic neuropathy in which morphological changes that occur at the optic nerve head and retinal nerve fiber layer are associated with functional deficit. Examining and monitoring the optic nerve head and the RNFL, structurally and functionally, is important for diagnosis and treatment. Apart from the topographical aspect, the relation between structural damage and functional damage also has a quantitative aspect.

Recently, the advent of computerized instruments such as confocal scanning laser ophthalmoscope (Heidelberg Retina Tomograph), Humphrey visual filed analyzer, and ultrasonic corneal pachymetry have introduced rapid and quantitative analysis of the optic nerve head and RNFL, visual field, and corneal thickness.

The cornea and sclera form the continuous collagen coat of the eye. In the posterior segment, the sclera perforates to form the lamina cribrosa through which retinal ganglion cell axon exit the eye. Changes in the sclera may be highly relevant in glaucoma, and scleral properties, such as elasticity and thickness may mirror those in the lamina. In experimental glaucoma there is acquired regional thinning in the posterior sclera that increase eye wall stress.[11] suggesting that individuals with thinner sclera may be at a higher risk from glaucomatous damage. However, there are no clinically available methods to measure posterior scleral or laminar thickness, but because of the close relation between the sclera and lamina, and by implication the lamina and cornea, many investigators have focused on the role of CCT in glaucoma. In other words, it may be that a thin central cornea is associated with a thin sclera, which in turn, is associated with a thin lamina. A thin central cornea is emerging as a major risk factor for severity of OHT and OAG.[1,5]

Anatomic and biometric evaluations of the optic disc and corneal thickness with correlation to various parameters, including sex, age, race, height, iris color, keratometry, anterior chamber depth, lens thickness, refraction, axial length, IOP, and various types of glaucoma have been made.[12]

The present study showed a significant correlation between thin CCT and optic disc area in POAG patients as also was reported by Jonas *et al*.[12] and Pakravan *et al*,[13] These finding may correspond with those in previous study, in which the horizontal and vertical corneal diameters, and the anterior corneal curvature radius correlated significantly and positively with the optic disc area, suggesting that eyes with a large optic disc have a thick cornea.[12] Gunvant *et al*.[14] reported that POAG eyes with thinner than average CCT values appear to be associated with larger and deeper optic disc cup.

Optic disc size influences the susceptibility of axonal damage in glaucoma. The particular propensity of the inferior and superior disc areas to excavation and axonal damage is associated with the higher lamina cribrosa pore-to-disc area ratio and thinner connective tissue support in these regions. However, with decreasing disc size, the pore-to-disc area ratio also decreases, providing greater tissue support,[15] thus thinner corneas may be a marker for more deformable discs, prone to the effects of increased IOP, whereas increased corneal thickness may simply indicate more rigid, resistant globes including optic disc lamina.[13]

In the current study, there was a significant correlation between thin CCT and neuroretinal rim area in POAG patients. This finding is consistent with Jonas *et al*.[12]

In patients with thin central cornea, the vasculature has become more damaged due to repetitive movements of the more complaint lamina. Subsequently, it may be less able to respond to IOP reduction with a beneficial increase in blood flow. Laminar sheet compression is common in glaucoma. These suggest an interrelationship between the topographic and vascular properties of the optic nerve head.[16]

In addition, thin CCT was associated with increased cup: disc ratio. This finding in agreement with Herndon who found that, for an increase of 10 μm of CCT, the vertical cup-disc ratio decreased by 0.008, and the horizontal cup-disc ratio decreased by 0.007.[1]

In the present study, there was a significant correlation between thin CCT and worsened mean deviation of visual field. This finding in agreement with Herndon *et al*.[1] who found that for an increase of 10μm of CCT, the mean deviation of visual field improved by 0.34 dB; for an increase of 10 years of age, the mean deviation of visual field worsened by 0.88 dB; for an increase of 1 mmHg of IOP, the mean deviation improved by 0.21 dB. Jonas *et al*.[12] found that progression of glaucomatous visual field loss was statistically independent of CCT. As, CCT measurements did not differ significantly between eyes with progression and eyes with stable visual field. This result in contrast to the OHTS, in which CCT was a significant risk factor for progression of ocular hypertension to POAG.[5] Also, shah *et al*.[17] who found that patients with thinner corneas initially present with a greater visual field defect, indicating that thin corneas may contribute to advanced glaucomatous damage at the time of diagnosis.

We found that patients with positive family history were not statistically significant associated with greater vertical cup:disc ratio (0.84) and more glaucoma medications (2.1). However, family history status had no effect on the mean deviation visual field defect. These findings are in a agreement with landers *et al*,[18] who found that a family history of POAG had no influence on the severity of visual field at diagnosis. They did find, however, that having a family history of glaucoma was associated with a better visual field at diagnosis in patients younger than 50 years but not in patients 50 years or older.

In the current study, there was significant correlation between thin CCT and increased number of glaucoma medications. This finding in agreement with Herndon *et al*.[1] who found that lower CCT was significantly associated with increased number of glaucoma medications; for an increase of 10 years of age, the number of medications increased by 0.23; for an increase of 1 diopter of spherical equivalent, the number of medications decreased by 0.06. Jonas *et al*.[12] found that no significant association between the use of topical B-blocker or topical carbonic anhydrase inhibitors and CCT. But, CCT tended to be greater in eyes that received latanoprost and smaller in eyes receiving pilocarpine.

In the current study, there was significant correlation between mean deviation visual field and disc area, rim area. These findings consistent with the results of Chauhan *et al*.[19] which showed that glaucomatous disc changes determined with scanning laser tomography occur more frequently than field changes. Most patients with field changes also had disc changes; however, less than half of those with disc changes had field changes.

Low CCT in POAG patient especially has clinical as well as statistical significance, since a patient’s glaucoma risk assessment may be directly affected by this decrease. The Early Manifest Glaucoma Trial demonstrated that glaucoma progression was lessened by 10% for every millimeter of mercury decrease in IOP, so adjusting IOP for decreased CCT may in fact alter glaucoma patient’s risk profile for progression. Thus, central corneal thickness was a strong predictive factor for the development of POAG, ever after adjusting for the effect of baseline age, IOP, cup-disc ratio, and visual field indices.[12]

## Conclusion

CCT is a significant predictor of glaucomatous damage as measured by cup:disc ratio, neuroretial rim, optic disc surface area, mean deviation of visual field in patients with POAG.

Measuring CCT in glaucoma patients my help identify those patients who are at high risk for developing severe glaucomatous sequelae, thus enabling the ophthalmologist to treat their disease early.
